# Wird die Rolle von psychischen Erkrankungen beim Suizid überbewertet?

**DOI:** 10.1007/s00103-021-03464-0

**Published:** 2021-12-07

**Authors:** Peter Brieger, Susanne Menzel, Johannes Hamann

**Affiliations:** 1 Akadem. Lehrkrankenhaus der LMU, kbo-Isar-Amper-Klinikum Region München, Vockestr. 72, 85540 Haar (bei München), Deutschland; 2 Klinik und Poliklinik für Psychiatrie und Psychotherapie, Klinikum rechts der Isar, Technische Universität München, München, Deutschland

**Keywords:** Suizidprävention, Universelle Prävention, Indizierte Prävention, Psychische Störung, Psychosoziale Faktoren, Suicide, Primary prevention, Secondary prevention, Mental disorders, Psychosocial factors

## Abstract

Die Aussage, dass Suizide zu 90 % Folge psychischer Erkrankungen sind, wird häufig in der wissenschaftlichen Literatur zitiert. Neuere Analysen und Kommentare ziehen das aber in Zweifel und betonen die Notwendigkeit, vielfältigere Ursachen für Suizidereignisse zu beachten, auch um die Prävention von Suiziden nicht auf das Erkennen und Behandeln psychischer Erkrankungen zu reduzieren. Das Ziel dieser Übersichtsarbeit ist die Darstellung und Bewertung wichtiger empirischer Befunde zu der Frage, ob die Rolle psychischer Störungen beim Suizid überbewertet wird.

Psychische Störungen erhöhen das Risiko eines Suizides um das bis zu 30- bis 50-Fache gegenüber der Allgemeinbevölkerung, dennoch wird dadurch nur ein Teil aller Suizide erklärt. Aus Beobachtungs- und Therapiestudien ergeben sich deutliche Hinweise, dass psychische Störungen nur ein Faktor unter mehreren sind, die zu Suizid führen. Eine Rolle spielen beispielsweise auch Beziehungsprobleme, Substanzmissbrauch, Belastungen durch schwere körperliche Erkrankungen, akute Krisen im Beruf, Probleme mit Finanzen und juristische Belastungen.

Suizidales Verhalten weist auf eine tiefe Unzufriedenheit hin, aber nicht notwendigerweise auf eine psychische Erkrankung. Viele Menschen mit einer psychischen Erkrankung zeigen kein suizidales Verhalten und nicht alle Menschen, die sich ihr Leben nehmen, haben eine psychische Erkrankung. Diese Erkenntnisse haben erhebliche Konsequenzen für die universale und indizierte Prävention von Suiziden.

## Abstract (en)

In the scientific literature, it is often said that 90% of all suicides are the consequences of a mental illness. Nevertheless, recent reports and comments cast doubt on this view and point out that such a limitation might hamper effective suicide prevention. In this overview we will outline and discuss important results on how often suicides are the consequence of mental illnesses and whether the association between mental illnesses and suicide might be overestimated.

Mental illnesses and especially affective disorders increase the risk of suicide by 30 to 50 times. Nevertheless, they explain only a certain percentage of all suicides. Observational and treatment studies indicate that mental illness is only one factor of several that lead to suicide. Among other factors are relationship problems, drug abuse, severe somatic illnesses, job problems, financial worries or juridical threats.

Suicidal behaviour is an indicator of deep unhappiness but not necessarily caused by a mental illness. Many persons with mental illnesses do not show suicidal behaviour. On the other hand, not all people who take their own lives have a mental illness. This has significant consequences for universal and indicated prevention of suicide.

## Hintergrund

Vielerorts ist zu lesen, dass mehr als 90 % aller Suizide durch psychische Erkrankungen bedingt seien. Auch das Bundesverfassungsgericht hat in seinem Urteil vom 26.02.2020 (2 BvR 2347/15) zur Sterbehilfe festgestellt: „Nach Einschätzung der sachkundigen Dritten bilden psychische Erkrankungen eine erhebliche Gefahr für eine freie Suizidentscheidung. Ihren Ausführungen zufolge liegen nach weltweit durchgeführten empirischen Untersuchungen in rund 90 % der tödlichen Suizidhandlungen psychische Störungen, insbesondere in Form einer Depression (in etwa 40–60 % der Fälle), vor. Depressionen, die häufig – selbst für Ärzte – schwer zu erkennen sind, führen etwa bei 20–25 % der Suizidenten zu einer eingeschränkten Einwilligungsfähigkeit“ (Randnummer 245, S. 71). Regelhaft werden die beiden Übersichtsarbeiten von Bertolote et al. [1, 2] zitiert, in denen anhand metaanalytischer Zusammenbringung vieler Studien (15.629 Suizide) gefolgert wird, dass es allgemein anerkannt sei, dass über 90 % derer, die Suizid begehen, zum Zeitpunkt des Todes eine psychiatrische Diagnose hatten. Aus dieser Perspektive heraus wird argumentiert, dass psychische Erkrankungen Ursache für die meisten Suizide seien und damit die beste Suizidprävention in der konsequenten Erkennung und Behandlung psychischer Erkrankungen in der Bevölkerung bestehe. Dies führt bis zu Überlegungen, dass die breite Gabe von selektiven Serotonin-Wiederaufnahme-Inhibitoren (SSRI) als antidepressive Medikation in der Bevölkerung die Zahl der Suizide reduziere und dass es eine inverse Korrelation zwischen der Verschreibung von Antidepressiva und der Zahl der Suizide gebe: Je besser die Versorgung der Bevölkerung mit Antidepressiva sei, umso geringer sei die Zahl der Suizide [3].

An der Sichtweise einer derart engen Assoziation zwischen psychischen Erkrankungen und Suiziden gibt es allerdings auch Zweifel und Kritik [4]. Zum einen ist es offenkundig, dass es den Suizid auch als Resultat einer gereiften, bilanzierenden Überlegung z. B. bei schwerer und finaler körperlicher Erkrankung ohne Vorliegen einer psychischen Erkrankung gibt. Wie häufig sich diese Fälle in der Gesamtzahl an Suiziden wiederfinden, ist allerdings unklar. Zum anderen kann aus der Tatsache, dass es bei Menschen mit einer bekannten psychischen Erkrankung zu einer krankhaften Einengung des Denkens hin zum Suizid kommen kann, nicht notwendigerweise darauf geschlossen werden, dass die psychische Erkrankung *ursächlich* für die Suizidalität sein muss. Vielmehr kann z. B. eine als unerträglich empfundene Lebenssituation den Hintergrund für eine akute psychische Krise mit daraus resultierender Suizidalität bilden. In derartigen Situationen spielt zwar eine Eingeschränktheit des Willens in der suizidalen Entscheidungssituation eine Rolle, wichtiger als eine rein medizinisch-psychiatrische Einschätzung und Einordnung erscheint hier aber der Blick auf Lebensverhältnisse, soziale Einbindung, Unterstützung in sensiblen Phasen von Lebensübergängen (Adoleszenz; Alter …) und Hilfen aller Art je nach Problematik (z. B. Palliativmedizin).

Kritisch hat Pompili [5] aus solchen Überlegungen heraus die Epidemiologie des Suizids diskutiert. Er fragte, wie es sein kann, dass weltweit Suizide in vielen Ländern zunehmen, wenn es doch – oft wirksame – wachsende Bestrebungen und Angebote im Bereich der Diagnostik und Behandlung psychischer Erkrankungen gäbe. Die US-amerikanische Regierungsbehörde Centers for Disease Control and Prevention (CDC) wies darauf hin, dass die These, Suizide seien vorrangig durch psychische Erkrankungen determiniert, nicht von ihren Daten gestützt wird und dass – gerade auch angesichts einer zunehmenden Zahl von Suiziden in den USA – diese Perspektive möglicherweise aktuell dazu führt, dass Suizidprävention partiell versagt, da sie an den falschen Punkten ansetzt [6]. Schließlich widersprechen auch die (vorläufig ausgewerteten) Auswirkungen der COVID-19-Pandemie einer monokausalen Erklärung von Suiziden: Während überwiegend davon ausgegangen wird, dass die psychiatrische Morbidität gestiegen ist [7], gibt es wenig Zweifel daran, dass die Zahl der Suizide in der Zeit der Pandemie keinesfalls zugenommen hat – vielmehr scheint sie, zumindest vorläufig, gesunken zu sein [8].

Wenn aber in Zweifel zu ziehen ist, dass Suizide vorrangig durch psychische Erkrankungen determiniert sind, dann ist auch die auf dieser Prämisse basierende Suizidprävention nicht so effektiv und zielgerichtet, wie das zu wünschen ist. Darauf hat auch Rüsch [9] hingewiesen, als er die Evidenz der Bündnisse gegen Depression („Alliance against Depression“) kritisch hinterfragte.

Letztlich lässt sich diese Debatte zugespitzt auf 2 Grundpositionen zurückführen:

- These 1: Suizide sind in der Regel direkte Folge psychischer Erkrankungen – und hier vor allem von affektiven Störungen^1^. Durch eine konsequente Therapie affektiver Störungen wird die Suizidrate reduziert.
- These 2: Selbsttötungen sind nicht monokausal durch psychische Krankheiten bedingt: Es kommen wesentliche Faktoren hinzu, die etwa im Bereich der psychosozialen Beziehungen, körperlichen Erkrankungen, Biografie und Persönlichkeit liegen. Solche Faktoren können auch ohne psychische Krankheit den Suizid bedingen.

Nachfolgend sollen Argumente für beide Grundpositionen dargestellt werden. Dabei wird sich zeigen, dass bei kritischer Reflexion der Fragestellung die Positionen gar nicht so weit auseinanderliegen, wie dies zunächst scheinen mag; wir hoffen vielmehr zeigen zu können, dass sie sich letztlich ergänzen.

## Psychische Erkrankungen sind die häufigste Ursache von Suiziden

Betrachtet man Kohorten von Menschen, bei denen eine psychiatrische Erkrankung diagnostiziert ist, dann zeigen diese eine deutlich erhöhte Häufigkeit von Suiziden. Eine umfassende Metaanalyse von Fu et al. [10] kommt auf der Basis von 41 Studien zu einer Suizidrate von Menschen mit schweren psychischen Erkrankungen von 313 pro 100.000 Personen. Dabei lag – basierend auf verschiedenen gepoolten Einzelstudien – die Suizidrate für Menschen, die an schwerer Depression leiden, bei 534 pro 100.000 Personen, die von Menschen mit Schizophrenie bei 352 und bei bipolarer Störung bei 237. Vergleicht man das mit den Suizidraten in der bundesdeutschen Allgemeinbevölkerung, die zwischen 10 und 15 auf 100.000 liegen (vgl. die Statistik von DESTATIS^2^), dann ist offensichtlich, dass die Suizidrate 25- bis 30-fach für schwere psychische Erkrankungen und für schwere Depressionen sogar 40- bis 50-fach erhöht ist.

Nordentoft et al. [11] kommen aus der Analyse schwedischer Registerdaten zu dem Ergebnis, dass im Verlauf von 36 Jahren einer Erkrankung das absolute Risiko für Suizid bei bipolarer Störung, unipolarer Depression oder Schizophrenie je nach Geschlecht und Diagnose zwischen 4,8 % und 7,8 % liegt – bei Menschen ohne psychische Störung betrug die Suizidhäufigkeit dagegen nur 0,7 % (Männer) bzw. 0,3 % (Frauen). Nahezu identische Zahlen berichtete auch die Metaanalyse von Moitra et al., die Studien aus nahezu allen Weltregionen einschließt [12]. Deswegen weisen Fu et al. [10] und Nordentoft et al. [11] darauf hin, dass frühere Zahlen zum Zusammenhang zwischen Suizid und psychischer Erkrankung, die bis heute zitiert werden und von einer Suizidhäufigkeit von 25 % und mehr bei affektiven Erkrankungen ausgehen, zu hoch sind und als widerlegt gelten sollten, so z. B. die Studie von Guze und Robins [13].

Sowohl Menschen mit als auch ohne psychische Erkrankungen können sich also das Leben nehmen, wobei psychische Erkrankungen unzweifelhaft das Suizidrisiko erhöhen. Aber selbst bei vergleichbarer Psychopathologie (z. B. einer wahnhaften Depression) begehen manche Menschen einen Suizid und andere nicht. Die Arbeitsgruppe um Baldessarini [14] berechnete einen Letalitätsindex für Suizidversuche und wies darauf hin, dass Menschen mit schweren affektiven Erkrankungen in ihren Suizidversuchen ein letaleres (eher zum Tode führendes) Vorgehen wählen als Menschen ohne psychische Erkrankungen. Es gibt in der Durchführung von Suizidversuchen Unterschiede zwischen psychisch erkrankten Menschen und solchen ohne psychische Erkrankung, wie es offenbar auch bei bestimmten psychischen Erkrankungen manche Suizidmethoden häufiger gibt [15]. Außerdem bestehen im Verlauf einer psychischen Erkrankung unterschiedliche Risikokonstellationen. Beispielsweise treten Suizide direkt nach Entlassung aus stationärer Behandlung deutlich gehäuft auf [16], wie auch während der Behandlung im psychiatrischen Krankenhaus [17, 18].

Es ist nur ein kleiner Teil der Menschen mit einer schweren psychischen und auch affektiven Erkrankung, der sich das Leben nimmt, – 90–95 % der Erkrankten tun dies nicht. Die Tatsache, an einer schweren psychischen Erkrankung zu leiden, erhöht allerdings das Risiko, sich das Leben zu nehmen, gegenüber der Allgemeinbevölkerung um den Faktor 10 bis 50. Es ist also unstrittig: Schwere psychische Erkrankungen erhöhen das Risiko, sich das Leben zu nehmen. Diese Personengruppe ist eine Zielgruppe für „indizierte Prävention“, also eine speziell für diese Risikogruppe vorgesehene Prävention.

## Viele Suizide werden von Menschen begangen, die an keiner psychischen Erkrankung leiden

Eine randomisiert kontrollierte Studie [19] zeigte, dass nach Suizidversuch die Kurzzeittherapie ASSIP (Attempted Suicide Short Intervention Program) in den folgenden 2 Jahren die Häufigkeit von weiteren Suizidversuchen um 80 % reduzieren konnte. Bei der Therapie stand das suizidale Verhalten im Fokus und nicht eine ggf. vorhandene psychische Störung. Die Bedeutung psychischer Störungen für die Suizidprävention wird durch die Ergebnisse also sehr stark relativiert.

Neuere Analysen von Regierungsbehörden in den USA und Italien gehen davon aus, dass höchstens 50 % aller Menschen, die sich das Leben nehmen, tatsächlich eine psychiatrische Diagnose aufweisen [6, 20]. Pompili [5] erklärt das folgendermaßen: „Suizidales Verhalten weist auf eine tiefe Unzufriedenheit hin, aber nicht notwendigerweise auf eine psychische Erkrankung. Viele Menschen mit einer psychischen Erkrankung zeigen kein suizidales Verhalten und nicht alle Menschen, die ihr Leben nehmen, haben eine psychische Erkrankung.“ Die neuere Forschung zu den Ursachen von Suizid hat – wie bereits dargestellt – eine Vielzahl weiterer Faktoren identifiziert. Dazu gehören u. a. Beziehungsprobleme, Substanzmissbrauch, Belastungen durch schwere körperliche Erkrankungen, akute Krisen im Beruf, Probleme mit Finanzen oder juristische Belastungen.

Auch in einer eigenen Untersuchung haben wir anhand von 626 Suizidfällen im Allgäu beobachtet, dass nur bei etwa der Hälfte der Suizidfälle eine psychische Störung bekannt oder anzunehmen war [21]. Hinzu kam ein Viertel, für das die Hinterbliebenen vorangegangene psychische Auffälligkeiten angaben. Hier kann man aber einerseits kritisch fragen, ob diese Auffälligkeiten nicht Ausdruck eben jener tiefen Unzufriedenheit waren, die zum Suizid geführt hat, und wo dabei genau die Grenze zwischen krank und gesund verläuft [22]. Bei einem weiteren Viertel waren aus den von der Polizei sehr gründlich geführten Akten keine Hinweise auf psychische Störungen erkenntlich.

Schließlich neigen Studien, die der Methode der „psychologische Autopsie“ [23] folgen, bei der Verhaltensweisen und Äußerungen der Suizidenten nachträglich bewertet werden, zu Verzerrungen (Bias), weil der Suizid zum Untersuchungszeitpunkt bereits bekannt ist und die Bewertung der vorliegenden Informationen entsprechend beeinflusst werden kann. Es kann daher vorkommen, dass die Fälle mit psychischen Erkrankungen überschätzt werden. Dies könnte auch auf die bereits angeführten Metaanalysen von Bertolote et al. [1, 2] zutreffen, die v. a. Studien zusammenfassten, die diese Methodik anwandten [5, 23, 24].

Es gibt also gute Evidenz, dass ein erheblicher Anteil der Suizident*innen nicht unter einer gesicherten psychischen Störung litt und dass anderslautende Befunde möglicherweise Resultat systematischer Bias sind. Auch für Menschen ohne psychische Erkrankung, aber mit hohem Risiko für einen Suizid durch spezifische psychosoziale Risikofaktoren sollte indizierte Prävention entwickelt und angeboten werden.

## Synthese: Suizide sind multifaktoriell bedingt und psychische Erkrankungen sind ein ganz wesentlicher Risikofaktor

Der oben zitierte Hinweis von Pompili [5], dass es nicht die psychische Störung ist, die zur Suizidhandlung führt, sondern bestimmte psychische Konstellationen, zu denen psychische Vorerkrankungen gehören können (aber nicht müssen), klingt zunächst einmal trivial, ist aber in Wirklichkeit aus unserer Sicht der Schlüssel zum Verständnis der vermeintlich sich widersprechenden Befunde: Es gibt bestimmte Konstellationen von inneren und/oder äußeren Ereignissen, die zu Hoffnungslosigkeit, Perspektivlosigkeit und unaushaltbarem psychischen Schmerz [25] führen. Es ist also weniger die – krankheitsbedingte – Suche nach dem Tod als die fehlende Lebensperspektive und der Schmerz, die zur Suizidhandlung führen. Schwere Depressionen, chronische bipolare Störungen, Schizophrenien und natürlich auch emotional instabile Persönlichkeitsstörungen sind hier Risikofaktoren, die zu solchen psychischen Phänomenen führen können. Es können aber auch andere innere und äußere Bedingungen in eine suizidale Krise münden, welche im Einzelfall in der psychiatrischen Diagnostik auch als „Anpassungsstörung“ dokumentiert werden könnte. Die suizidale Krise wäre somit auch in ICD-11 oder DSM(Diagnostic and Statistical Manual of Mental Disorders)-V als Diagnose abbildbar, ohne notwendigerweise als „Erkrankung“ im eigentlichen Sinn zu gelten. Denn um eine eigentliche Erkrankung im Sinne von Mario Maj [26], die man präventiv durch eine z. B. medikamentöse Behandlung beheben und so dem Suizid vorbeugen könnte, handelt es sich dabei nicht. Die konsequente Behandlung einer psychischen Grunderkrankung kann zwar auch das Risiko für Suizide reduzieren, ersetzt aber nicht die Auseinandersetzung mit der subjektiven Erfahrung einer suizidalen Krise im Rahmen eines patientenzentrierten Vorgehens [27].

## Fazit

Es besteht also ein enger Zusammenhang zwischen psychischen Erkrankungen und Suizid und gleichzeitig besteht die Gefahr, dass die Einengung der Suizidprävention auf die Diagnostik und Behandlung psychiatrischer Erkrankungen zu kurz greift; eine daran geknüpfte Konzentration von finanziellen Ressourcen auf diese Maßnahmen gefährdet u. U. die Verbesserung und Erweiterung suizidpräventiver Bemühungen auf verschiedenen gesellschaftlichen Ebenen (Prävention für bestimmte vulnerable Gruppen, z. B. Schüler*innen, alte Menschen oder Wohnungslose jeweils ohne psychiatrischen Hintergrund; Krisenversorgung; bauliche Suizidprävention). Es steht zu befürchten, dass die stetige Zunahme von Suiziden in manchen Regionen außerhalb Deutschlands darauf zurückzuführen ist, dass Suizidprävention nur eingeschränkt verfügbar ist oder den falschen Fokus wählt. Suizidprävention sollte breiter aufgestellt sein und die Vielzahl der Phänomene in den Fokus nehmen, die zur Suizidalität führen können. Dies können neben einer psychiatrischen Morbidität auch Verzweiflung und Hoffnungslosigkeit in einer krisenhaften Lebenssituation, psychischer oder körperlicher Schmerz, fehlende soziale Kohäsion, Einsamkeit, Angst vor Strafe, Scham und manches andere sein.

Solche Bedingungen können das menschliche Dasein so sehr zerrütten, dass es aus der empfundenen Perspektivlosigkeit heraus zu einer suizidalen Handlung kommt. Entsprechend bedarf es Krisenkonzepten, die über eine rein medizinische Behandlung hinausgehen [28]. Dem muss erfolgreiche Suizidprävention auf mehreren Ebenen Rechnung tragen. Diese Ausführungen gelten ebenso für die Nachsorge von Suizidversuchen als indizierte Prävention. Auch dort sind weitergehende Konzepte wichtig, die über eine medikamentöse Therapie hinausgehen – wie es beispielsweise der Beitrag von Teismann und Gysin-Maillart [27] in diesem Themenheft darstellt. Schließlich bedarf es auch Konzepte der selektiven Prävention durch eine bessere Identifikation von Risikogruppen, denen entsprechende Hilfen angeboten werden (z. B. durch einen Krisendienst). Universelle Prävention gelingt durch eine Verbesserung der allgemeinen psychiatrisch-medizinischen Versorgung wie auch durch Verbesserung der Lebens- und Arbeitsbedingungen. So gelingt in der Summe durch ein besseres Verständnis dafür, wer sich mit welchen Motiven und Hintergründen das Leben nimmt, auch eine bessere Suizidprävention.

## Footnotes

1 Als affektive Störungen werden Erkrankungen bezeichnet, die mit einer Veränderung der Stimmungs- und Gemütslage einhergehen, z. B. Depressionen und bipolare Störungen. Sie sind im Kapitel F3 der ICD(International Statistical Classification of Diseases and Related Health Problems)-10 aufgeführt.

2 https://www.destatis.de/DE/Themen/Gesellschaft-mwelt/Gesundheit/Todesursachen/Tabellen/suizide.html.
